# Acute myocardial infarction and cardiac arrest induced by oxymetazoline nasal spray overdose: a case report

**DOI:** 10.1093/ehjcr/ytag176

**Published:** 2026-03-10

**Authors:** Mhd Baraa Habib, Firas Hamsho, Anas A Ashour, Hiba Habib, Mohammed Mohsen

**Affiliations:** Cardiology Department, Heart Hospital, Hamad Medical Corporation, 3050 Doha, Qatar; Cardiology Department, Heart Hospital, Hamad Medical Corporation, 3050 Doha, Qatar; Cardiology Department, Heart Hospital, Hamad Medical Corporation, 3050 Doha, Qatar; Internal Medicine Department, Damascus University Hospital, 10769 Damascus, Syria; Cardiology Department, Heart Hospital, Hamad Medical Corporation, 3050 Doha, Qatar

**Keywords:** Oxymetazoline, Nasal spray, Coronary vasospasm, Acute myocardial infarction, Cardiac arrest, Case report

## Abstract

**Background:**

Oxymetazoline nasal spray is a widely used over-the-counter sympathomimetic decongestant. While generally safe at therapeutic doses, overdose can result in systemic vasoconstriction and potentially life-threatening cardiovascular complications.

**Case summary:**

We report the case of a previously healthy 27-year-old man who presented after an out-of-hospital cardiac arrest following excessive use of oxymetazoline nasal spray. He was found in ventricular fibrillation and required cardiopulmonary resuscitation and defibrillation. Post-resuscitation electrocardiogram showed transient ST-segment elevations, and cardiac biomarkers were markedly elevated. However, emergent coronary angiography revealed normal coronary arteries. Comprehensive cardiac evaluation, including cardiac magnetic resonance imaging and electrophysiologic studies, excluded myocardial infarction, myocarditis, myocardial fibrosis, and Brugada syndrome. The patient experienced prolonged neurological recovery but improved with rehabilitation.

**Discussion:**

Oxymetazoline overdose should be considered a potential cause of acute coronary syndrome, malignant arrhythmias, and cardiac arrest, even in young individuals without pre-existing cardiovascular disease. Clinician awareness and patient education on the safe use of sympathomimetic agents are essential for prevention.

Learning points

- Oxymetazoline overdose can potentially cause life-threatening cardiovascular events, including coronary vasospasm, acute myocardial infarction, and cardiac arrest, even in young, healthy individuals.
- Sympathomimetic-induced ST-elevation with normal coronary arteries should prompt consideration of vasospastic mechanisms, especially with a history of decongestant use.
- Patient education on the safe use of over-the-counter nasal decongestants is critical to prevent serious systemic toxicity and adverse outcomes.

## Introduction

Oxymetazoline hydrochloride is an over-the-counter α-adrenergic agonist commonly used as a topical nasal decongestant. Its primary mechanism involves selective activation of α_1_-adrenergic receptors, leading to vasoconstriction of the nasal mucosa and consequent relief of congestion.^1^ At recommended doses, systemic absorption is typically minimal due to its topical administration and poor bioavailability. However, excessive or prolonged use, particularly at doses exceeding therapeutic recommendations, can result in systemic absorption and significant sympathomimetic toxicity, including severe hypertension, syncope, and cardiovascular complications due to widespread vasospasm.^2–7^

Although cardiovascular adverse events associated with nasal decongestants are relatively rare, they can be clinically significant and potentially life-threatening.^8,9^ These risks may be underrecognized, given the widespread availability and perceived safety of these medications. In this report, we describe a case of acute myocardial infarction (MI) complicated by cardiac arrest following a substantial overdose of oxymetazoline nasal spray. This case underscores the potential for life-threatening cardiovascular toxicity associated with excessive use of topical sympathomimetics and highlights the need for greater awareness regarding their systemic effects.

## Summary figure

**Figure:**
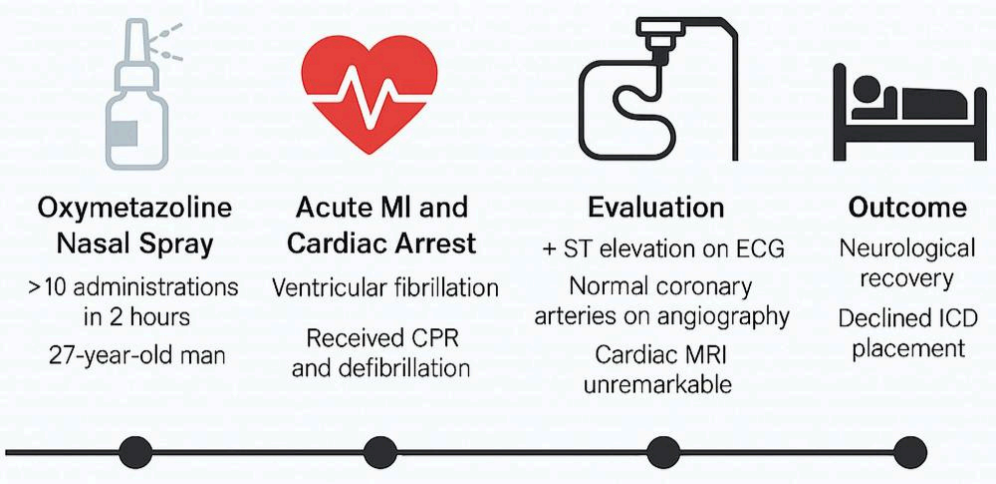


## Case report

A previously healthy 27-year-old man was brought to the emergency department following an out-of-hospital cardiac arrest. According to his roommate, the patient had been experiencing flu-like symptoms and nasal congestion for 3 days. On the day of admission, he reportedly used an over-the-counter oxymetazoline hydrochloride nasal spray more than 10 times within a 2-h period. Shortly thereafter, he developed sudden, severe chest pain and shortness of breath, followed by collapse and unresponsiveness.

**Figure 1:**
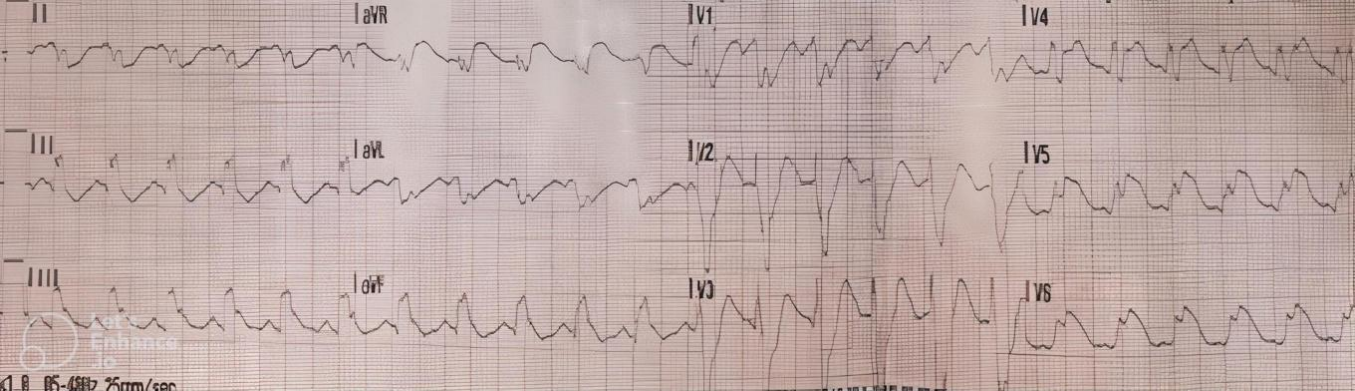
Initial electrocardiogram by emergency medical services showing ST-elevation in leads V4–V6 with an intraventricular block that was suspicious for myocardial ischaemia as a case of the cardiac arrest.

Upon arrival of emergency medical services, the patient was found to be pulseless, and the electrocardiogram (ECG) showed ST elevation in leads V4–V6 with an intraventricular block (*Figure 1*). Cardiopulmonary resuscitation (CPR) was initiated, and return of spontaneous circulation (ROSC) was achieved after two cycles of CPR and one 200 joule defibrillation shock. He subsequently deteriorated into pulseless electrical activity and required two additional cycles of CPR and intravenous adrenaline before ROSC was achieved. Due to inadequate respiratory effort, he was intubated in the field.

Further history obtained later revealed no known chronic medical conditions. The patient was not taking any regular medications and had no history of smoking, alcohol consumption, or recreational drug use. There was no family history of sudden cardiac death. He was unemployed and had not travelled recently.

**Figure 2:**
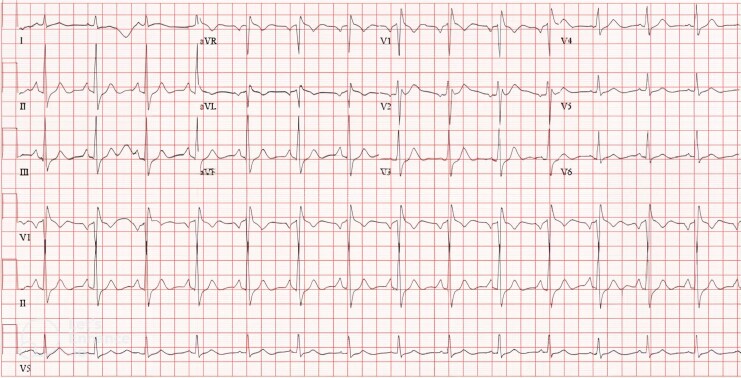
Electrocardiogram upon arrival at the hospital showing resolution of the ST-elevation that was previously seen during cardiac arrest.

On arrival at the emergency department, the patient remained unconscious but had stable vital signs. An ECG revealed resolution of ST-segment elevation in the lateral leads (*Figure 2*). Laboratory investigations showed markedly elevated high-sensitivity troponin levels (396, 1908, and 642 ng/L; reference range < 15 ng/L), potassium of 3.5 mmol/L (3.5–5.3 mmol/L), and respiratory acidosis with pH of 7.25 (7.32–7.42), PaCO_2_ of 63 mmHg (41–51 mmHg), and bicarbonate of 27.6 mmol/L (23–29 mmol/L), along with elevated serum lactate of 3.4 mmol/L (0.5–2.2 mmol/L). Computed tomography (CT) of the brain and CT pulmonary angiogram were unremarkable.

**Figure 3:**
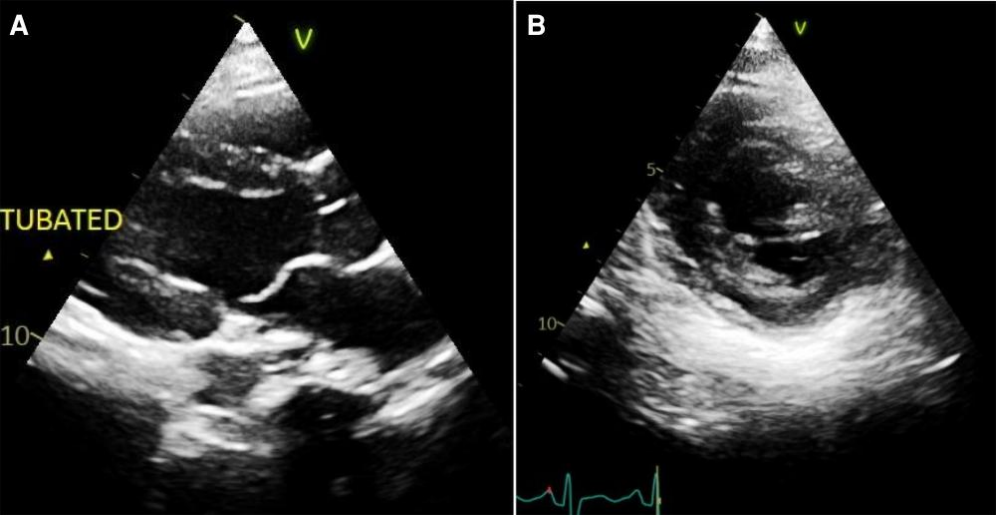
Transthoracic echocardiography demonstrating normal cardiac anatomy and function without evidence of structural heart disease: (*A*) parasternal long-axis view and (*B*) parasternal short-axis view.

**Figure 4:**
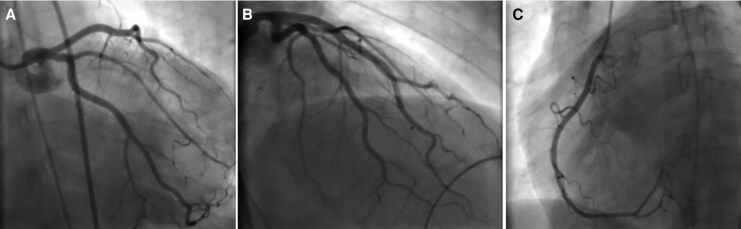
Coronary angiography (CAG) demonstrating normal coronary arteries and ruling out Type 1 myocardial infarction: (*A*) left coronary artery system in the anteroposterior (AP) caudal view, (*B*) left coronary artery system in the right anterior oblique (RAO) cranial view, and (*C*) right coronary artery in the left anterior oblique (LAO) view.

Bedside focused cardiac ultrasound demonstrated good left ventricular contractility without pericardial effusion. Transthoracic echocardiography confirmed a left ventricular ejection fraction (LVEF) of 53% with normal right ventricular function (*Figure 3*). Urgent coronary angiography revealed angiographically normal coronary arteries with no evidence of obstructive coronary artery disease (*Figure 4*).

The patient was admitted to the cardiac intensive care unit and subsequently underwent tracheostomy after 14 days for prolonged ventilatory support. Neurologic assessment raised concern for hypoxic–ischaemic encephalopathy. However, he gradually demonstrated neurological improvement within 20 days of admission, was successfully decannulated, and was transferred to the cardiology ward in stable condition.

Cardiac magnetic resonance imaging (MRI) performed during hospitalization showed normal biventricular size and function (LVEF 61%, right ventricular ejection fraction 49%) without evidence of myocardial oedema, fibrosis, infarction, or cardiomyopathy (see Supplementary material online, *Figure S1*). During the admission, the patient developed a transient ECG finding suggestive of a Brugada-like pattern (see Supplementary material online, *Figure S2*). Consequently, an electrophysiology study and an ajmaline challenge were performed to evaluate for Brugada syndrome and other inducible ventricular arrhythmias as potential causes of the cardiac arrest; neither test demonstrated Brugada syndrome or inducible ventricular arrhythmias.

In accordance with current guidelines, implantation of an implantable cardioverter-defibrillator (ICD) was recommended for secondary prevention of sudden cardiac death.^8^ After a detailed discussion of the indications, risks, and benefits, the patient elected to defer ICD placement at that time.

He was counselled to avoid the use of over-the-counter sympathomimetic agents, particularly oxymetazoline, and was referred for inpatient rehabilitation with plans for ongoing outpatient cardiology follow-up.

## Discussion

Oxymetazoline hydrochloride is a topical nasal decongestant in the imidazoline class, widely recognized for its selective α-adrenergic agonist properties. It primarily acts via stimulation of α_1_-adrenergic receptors, leading to vasoconstriction of nasal mucosal blood vessels and consequent reduction in oedema and nasal congestion.^1^ While systemic absorption is minimal when used at therapeutic doses, significant overdose can result in systemic sympathomimetic effects, such as severe vasoconstriction, hypertension, arrhythmias, and, in rare cases, MI.^2–7^ The oxymetazoline nasal spray has a concentration of 0.5 mg/mL and typically delivers ∼50–100 µL of solution per actuation, corresponding to roughly 25–50 µg of oxymetazoline per spray. As it was reported that the patient used the spray more than 10 times, the estimated exposure was ∼250–500 µg of oxymetazoline.

This case highlights a rare but severe potential cardiovascular complication of oxymetazoline overdose—coronary vasospasm resulting in acute MI and cardiac arrest. The patient’s presentation was consistent with an acute coronary syndrome, as evidenced by acute chest pain, ST-segment elevations on ECG, and markedly elevated troponin levels. However, the absence of obstructive lesions on coronary angiography strongly supports a vasospastic mechanism rather than atherosclerotic coronary artery disease. This aligns with prior reports of sympathomimetic-induced vasospasm presenting with elevated cardiac biomarkers and ischaemic ECG changes, but with angiographically normal coronary arteries.^9,10^ Rajpal *et al*. (2014)^9^ described a similar case involving a 64-year-old woman who developed non–ST-elevation MI following oxymetazoline use, with subsequent angiography revealing normal coronary arteries. Another report documented a case of sinus arrest in a 2-year-old child following severe hypertension and reflex bradycardia caused by intraoperative administration of oxymetazoline, highlighting the profound cardiovascular effects even in paediatric populations.^10^  *Table 1* summarizes case reports and case series describing cardiac arrest, cardiac collapse, or myocardial infarction following nasal decongestant use.

**Table 1:** Summary of reported cases in the literature of cardiac arrest or cardiac collapse associated with nasal decongestants

#	Year	Author(s)	Drug/route/concentration	Patient (age/sex/context)	Cardiac event described	Outcome/comments	Reference
1	1995	Thrush DN	Oxymetazoline nasal spray (0.025%)	2-year-old child, intraoperative during nasal endoscopy	Severe hypertension → reflex bradycardia → sinus arrest/cardiac arrest requiring CPR and atropine	Survived after resuscitation	Thrush DN. Cardiac arrest after oxymetazoline nasal spray. J Clin Anesth. 1995;7(6):512–514. doi:10.1016/0952-8180(95)00060-u
2	2015	Kouzegaran S	Phenylephrine (topical intranasal pledgets)	9-year-old girl, during adenoidectomy	Intraoperative cardiac arrest shortly after phenylephrine use	Resuscitated/recovered	Kouzegaran S. A Case of Cardiac Arrest after Topical Phenylephrine. J Pediatr Pharm Pract. 2015/03/01. DOI: 10.22038/ijp.2015.4061.
3	2010	Kaye Ad *et al*.	Phenylephrine topical/intranasal	21-year-old man, intraoperative	Pulseless electrical activity (PEA) cardiac arrest immediately after topical phenylephrine use	Successful resuscitation with epinephrine; patient recovered fully	Kaye, A. D., Sabartinelli, A. L., Kaye, A. M., Holtzman, A. M., & Samm, P. L. Intraoperative pulseless electrical activity and acute cardiogenic shock after administration of phenylephrine, epinephrine, and ketamine. Ochsner Journal, 10(3), 205–209.
4	2014	Rajpal S, Morris LA, Akkus N	Oxymetazoline nasal spray (0.05%)	64-year-old woman, outpatient setting	Non-ST-elevation myocardial infarction (prolonged chest pain, biomarker rise) following spray use	Survived; underwent angiography (normal coronaries)	Rajpal S, Morris LA, Akkus NI. Non-ST-elevation myocardial infarction with the use of oxymetazoline nasal spray. Rev Port Cardiol. 2014;33(1):51.e1–51.e4. doi:10.1016/j.repc.2013.07.011

The pathophysiology of oxymetazoline-induced coronary vasospasm likely involves excessive activation of α-adrenergic receptors on coronary smooth muscle, resulting in intense vasoconstriction, reduced myocardial perfusion, and potential ischaemia or infarction.^4,5,7,11–13^ In addition to a direct ischaemic effect from systemic α-adrenergic stimulation, other non-atherothrombotic mechanisms may contribute to acute myocardial injury in the absence of obstructive coronary disease. Stress (Takotsubo) cardiomyopathy is characterized by transient left ventricular dysfunction triggered by a surge in catecholamines, with proposed mechanisms including microvascular dysfunction, multivessel coronary spasm, and direct catecholamine-induced myocardial stunning and toxicity. These processes can produce myocardial ischaemia and troponin elevation despite patent epicardial arteries. Although our case did not demonstrate typical ventricular wall motion abnormalities of stress cardiomyopathy, these alternative mechanisms highlight the potential role of catecholamine-mediated vasomotor and microvascular dysfunction in precipitating myocardial injury in the setting of excessive sympathomimetic exposure.^14^ While cardiovascular complications from oxymetazoline are exceedingly rare, this case underscores the need for awareness of the drug’s systemic toxicity especially when used in excess.

Cardiac MRI performed during recovery demonstrated normal biventricular structure and function without evidence of myocardial fibrosis, oedema, or infarction, effectively ruling out myocarditis or structural heart disease. The reversibility of ECG changes and resolution of regional wall-motion abnormalities further support a transient, vasospastic aetiology. Additionally, the absence of angiographic spasm or fixed stenosis, combined with complete resolution of ST-segment elevations, as well as absence of further chest pain or ECG changes, supported a transient event, possibly triggered by excessive sympathomimetic stimulation from oxymetazoline rather than a chronic vasospastic condition.

Several differential diagnoses were considered in this case. Illicit stimulant use (e.g. cocaine and amphetamines), known to induce coronary vasospasm and arrhythmias via catecholaminergic surge, was excluded based on the patient’s history and negative toxicology screening.^11–13^ Brugada syndrome, considered due to transient ECG changes, was ruled out through an electrophysiological study and ajmaline challenge.^15,16^ Obstructive coronary artery disease and structural heart conditions were ruled out with normal coronary angiography, echocardiography, and cardiac MRI. Other systemic causes of cardiac arrest, such as pulmonary embolism, were ruled out by CT pulmonary angiogram. Since it was not definitively confirmed that oxymetazoline caused the cardiac arrest, the recommendation for an ICD as a secondary prevention of sudden cardiac death was made in line with current guidelines,^8^ though the patient opted against device implantation. Calcium channel blockers or nitrates were not initiated because there was no angiographic or clinical evidence of ongoing vasospasm, and no recurrent ischaemic episodes occurred during the hospital stay. Management focused on eliminating the precipitating factor—excessive sympathomimetic nasal decongestant use—and close monitoring for arrhythmic recurrence.

This report describes a temporal association between excessive oxymetazoline use and acute myocardial infarction with cardiac arrest; however, a definitive causal link cannot be established. Quantitative serum oxymetazoline levels were not available, as validated assays are not routinely accessible in clinical practice, precluding objective confirmation of systemic exposure. In addition, a coronary provocation test could have helped demonstrate an underlying vasospastic disease. However, it was not done as the patient was recently recovering from a cardiac arrest, and the procedure carries a potential risk of inducing malignant arrhythmias or recurrent ischaemia. Nevertheless, from a public health and preventive perspective, this case highlights the need for enhanced education on the safe use of over-the-counter medications, particularly sympathomimetic nasal sprays. Young adults may underestimate the potential systemic effects of these agents. Clinicians should maintain a high index of suspicion for sympathomimetic toxicity in patients presenting with acute coronary syndrome-like symptoms in the absence of coronary disease. Prompt recognition and management can mitigate serious outcomes.

## Conclusion

Oxymetazoline nasal spray, although commonly perceived as safe, could lead to severe systemic cardiovascular complications when overdosed. Clinicians should maintain vigilance for sympathomimetic-induced coronary vasospasm as a rare but significant cause of acute coronary syndrome and sudden cardiac arrest. Patient education on appropriate use and potential risks of over-the-counter sympathomimetics is critical to preventing such adverse events.

## Supplementary Material

ytag176_Supplementary_Data

## Data Availability

Data sharing is not applicable to this article as no datasets were generated or analysed during the current study.

## Lead author biography

Dr Mhd Baraa Habib is a cardiology fellow at Hamad Medical Corporation in Doha, Qatar, where he began his training in 2023 following completion of his internal medicine residency at the same institution. He is certified by the Arab Board, Qatari Board, and MRCP (UK) and has a strong academic record. Dr Habib has authored over 60 publications, including case reports, research articles, and conference presentations. His research focuses on cardiovascular imaging, cardiogenic shock, and pulmonary hypertension, and he is actively involved in research.

## Notes

**Consent:** Written informed consent was obtained from the patient to publish this report in accordance with the journal’s patient consent policy. The case was approved for publication by Hamad Medical Corporation IRB with a protocol number MRC-04-24-319.
