# Supplementary figures and images for: Acute myocardial infarction and cardiac arrest induced by oxymetazoline nasal spray overdose: a case report
Source: Eur Heart J Case Rep. 2026 Mar 10;10(3):ytag176. doi: 10.1093/ehjcr/ytag176 (PMC13019283; doi:10.1093/ehjcr/ytag176)

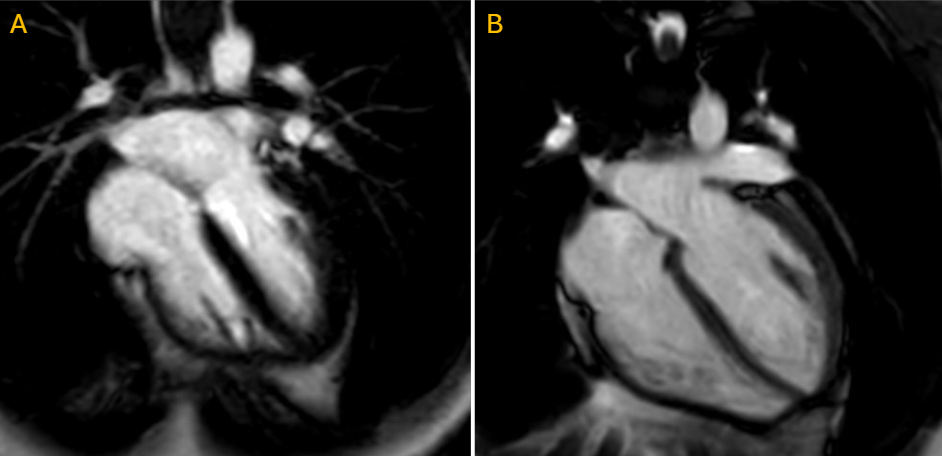

Supplement: ytag176_Supplementary_Data [file ytag176_supplementary_data.zip › 25-00689_S1.png]

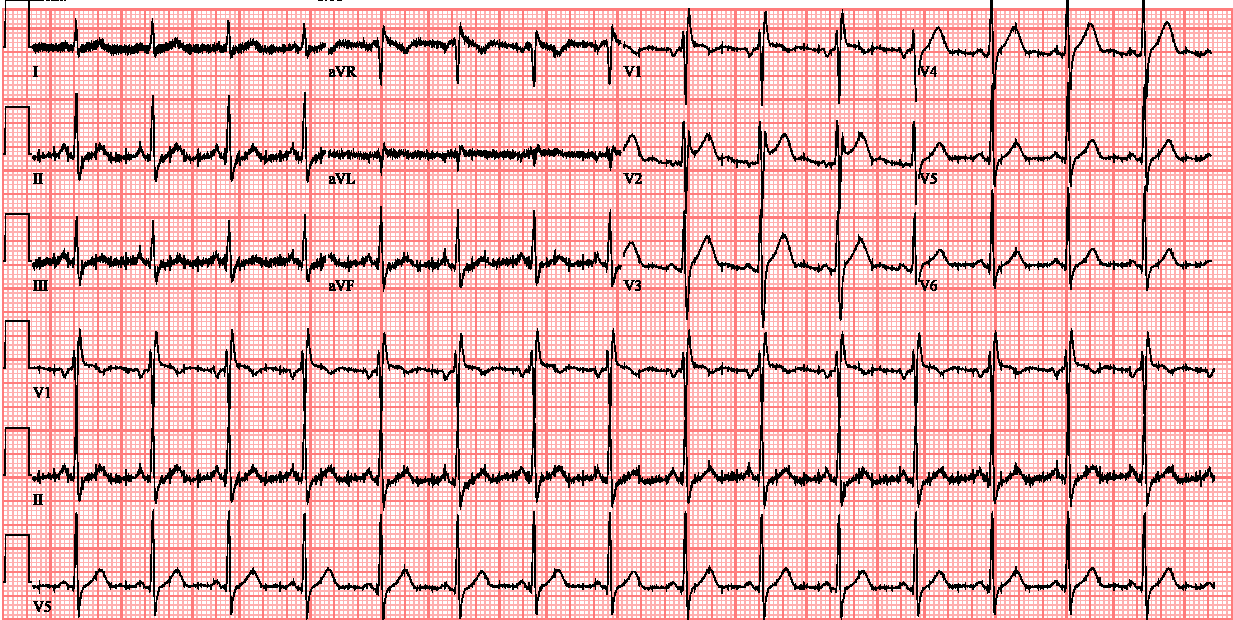

Supplement: ytag176_Supplementary_Data [file ytag176_supplementary_data.zip › Figure S2.tif]
